# Current Iodine Nutrition Status and Morbidity of Thyroid Nodules in Mainland China in the Past 20 Years

**DOI:** 10.1007/s12011-020-02565-2

**Published:** 2021-02-13

**Authors:** Xin Liu, Jian Sun, Wei Fang, Yanguo Xu, Zizhao Zhu, Yazhuo Liu

**Affiliations:** 1 Department of Critical Care Medicine, The Affiliated Hospital of Qingdao University, Qingdao, Shandong China; 2 Department of Neurosurgery, The Affiliated Hospital of Qingdao University, Qingdao, Shandong China; 3 Department of Critical Care Medicine, The Affiliated Suzhou Hospital of Nanjing Medical University, Suzhou, Jiangsu China; 4 Department of General Surgery, The Sixth People’s Hospital of Shenyang, Shenyang, China; 5 Department of Clinical Nutrition and Metabolism, The Affiliated Zhongshan Hospital of Dalian University, Dalian, Liaoning China

**Keywords:** Thyroid nodules, Urinary iodine concentration, Morbidity, China

## Abstract

The aim of this study was to comprehensively assess the prevalence of goiter and thyroid nodules (TNs) in relation to China’s iodine nutrition level over the past 20 years and provide an effective reference for developing health policies. PubMed, EMBASE, Chinese National Knowledge Infrastructure, Chongqing VIP, and Chinese Wan Fang databases were searched for relevant studies from Jan 1996 to Feb 2020. Two reviewers extracted valid data from the eligible citations to determine the morbidity of TNs in different urinary iodine concentrations (UICs) and in patients of different genders, of different ages, who live in different geographic regions, and who live at different altitudes, as well as the *P* values of interactions between groups. There were 26 articles (34 studies) included in this analysis. The overall morbidity of TNs in mainland China was 23.4%. Morbidity was higher in urban areas (*P* < 0.001) than in rural and mixed areas. Coastal areas (*P* < 0.001), female patients (*P* < 0.001), high-altitude areas (*P* < 0.001), and residence in south China (*P* < 0.001) were all associated with higher morbidity of TNs. The lowest morbidity value of TNs, 16%, was in the more-than-adequate iodine subgroup. The highest morbidity, 27.2%, was in the adequate iodine subgroup. The morbidity of TNs increases with age, and women are more likely to have TNs. We also need to perform more epidemiological studies, and in the future, we should cultivate better understanding of the relationship between other thyroid diseases and provide more comprehensive and useful information for other researchers.

Iodine is a trace element that plays an important role in the synthesis of the thyroid hormones thyroxin (T4) and triiodothyronine (T3), which are essential for life [1]. Normally, it involves impermanent shutdown of thyroid hormone synthesis in response to supraphysiologic iodine exposure, known as the acute Wolff–Chaikoff effect [2]. In 1990, the World Health Organization (WHO) found that 2.2 billion people in 130 countries were at risk of iodine deficiency disorder (IDD). Adequate intake of iodine is vitally important for synthesizing thyroid hormones and minimizing the risk of thyroid disease in adults. In light of WHO recommendations, urinary iodine concentration (UIC) was used to estimate iodine status in populations, which is recommended to be between 100 and 199 μg/L in schoolchildren and adults. There is also great concern that excess iodine, like iodine deficiency, may have negative effects on thyroid function [3]. A 5-year prospective survey performed in China shows that excess iodine can induce and promote the incidence and development of hypothyroidism and autoimmune thyroiditis [4].

Thyroid nodules (TNs) are independent, structurally separate neoplasms within the thyroid gland [5], with 3–7% morbidity by palpation [6]; 20–76% are found by ultrasound [7], and 8.2–65% are not found until autopsy [8, 9]. In recent years, an increasing incidence of thyroid carcinoma has been reported in many countries [10–13]. The morbidity of thyroid carcinoma in patients with thyroid nodules can be as high as 15% [14].

China implemented universal salt iodization (USI) regulations to prevent IDDs in 1996, involving all 31 provinces of mainland China. In 2011, the standard of salt iodization concentration in China was adjusted to household salt iodine content of 20–30 mg/kg, and provinces were allowed to choose salt iodization concentrations according to local conditions. During the two decades in which USI standards have been used, the population of China has been consecutively exposed to an iodine nutrition status of excessive iodine intake from 1996 to 2001, more-than-adequate iodine intake from 2002 to 2011, and adequate iodine intake from 2012 to 2016 [15].

Thus, we here present a systematic review and meta-analysis to analyze the morbidity of TNs after enforcement of the USI project in mainland China over the course of two decades.

## Materials and Methods

### Literature Search Strategy

We artificially retrieved all of the literature concerning population-based studies on the morbidity of thyroid nodules from January 1996 to September 2020 making use of the PubMed, EMBASE, Chinese National Knowledge Infrastructure, Chongqing VIP, and Chinese Wan Fang databases. The keywords “thyroid disorder(s),” “thyroid nodule(s),” “TN(s),” and “iodine” or the terms “prevalence(s)” or “incidence(s),” or “epidemiology” and “China” or “Chinese” were used to search for relevant studies. We also checked the reference list of identified studies in order to find more additional studies.

### Selection Criteria

Our inclusion criteria were as follows: (1) the people were from stochastic community-oriented samples rather than voluntary acceptors or patients undergoing routine medical examinations; (2) the research design was population-based rather than hospital-based; (3) research results covered enough information (e.g., research geographic region, survey methodology, number of cases, sample size, diagnostic criteria, and urinary iodine concentration).

Studies were removed if they met any of the following exclusion criteria: (1) case reports or reviews; (2) the people had any relevant sickness or took drugs or therapy known to influence thyroid structure or function; (3) the study centered on people in a specific subpopulation (such as smokers or gestational women) or who shared a specific career; (4) they were the same studies republished.

### Data Extraction

**Fig. 1:**
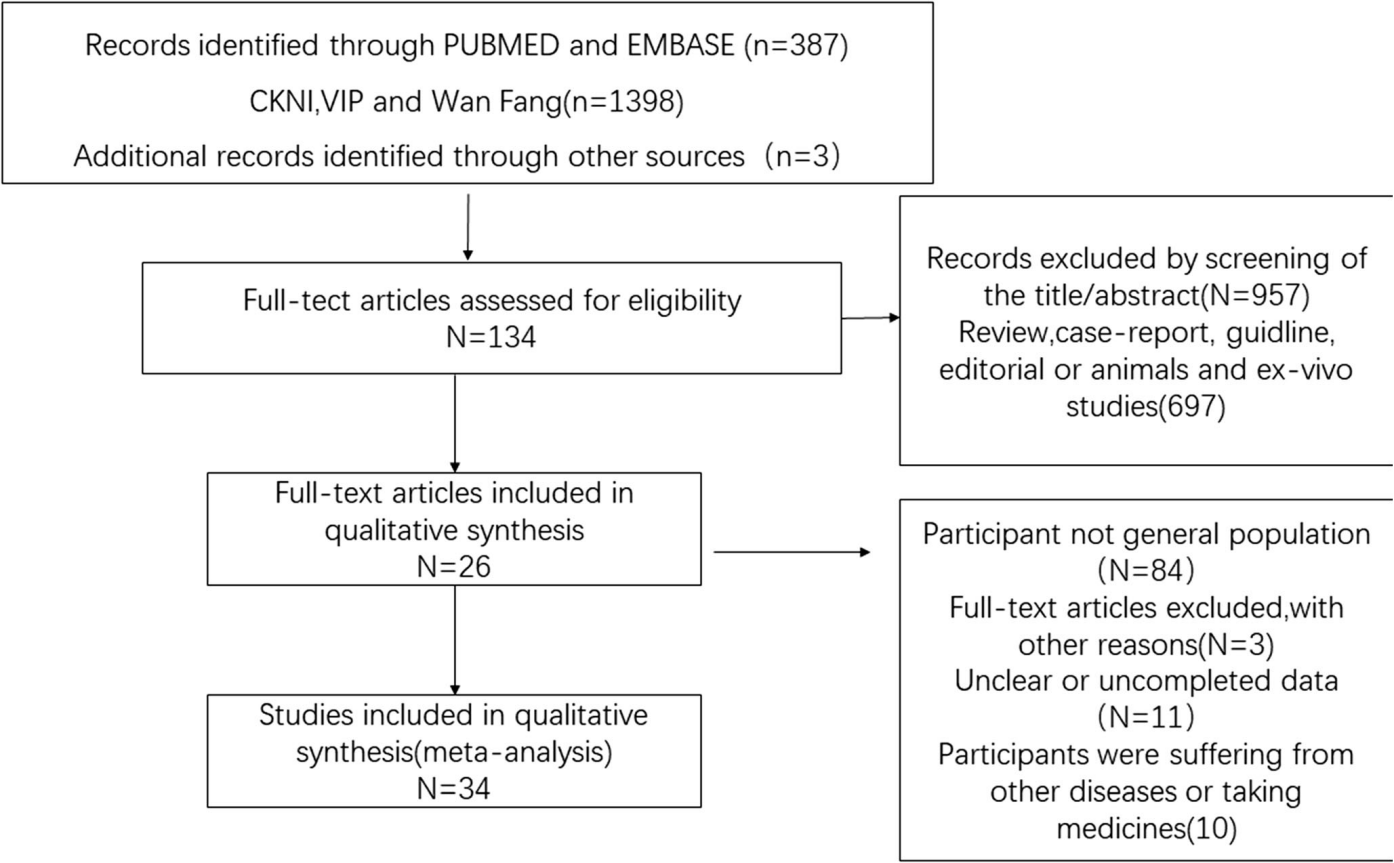
Flow diagram of the literature-search process

Two reviewers separately extracted information, specifically the first author, the date of publication, the starting year of the work, age of the participants, geographic region, sample size, outcomes, and prevalence. The literature-retrieval process is shown in Fig. 1. Any differences were resolved by consensus. In our study, the median UIC was used to sort subjects into four subgroups: insufficient group (median UIC ≤ 99 μg/L); adequate group (median UIC between 100 and 199 μg/L); more-than-adequate group (median UIC between 200 and 299 μg/L); excessive group (median UIC ≥ 300 μg/L).

### Statistical Analysis

We summarized the frequency of TNs along with 95% confidence intervals (CIs) to evaluate the morbidity of TNs in mainland China. The *χ*^2^-based 푄 test and the 퐼^2^ test were used to calculate the heterogeneity of the studies. The low, moderate, and high levels of heterogeneity were set as 25%, 50%, and 75%, respectively [16]. We used a random-effects meta-analysis model to replace the fixed-effects model. We used the Egger’s test to estimate for publication bias (*P* < .05 was considered of statistical significance) when the level of heterogeneity was moderate or high. Stata Version 16.0 (Stata Corp LP, TX, USA) was used to perform meta-analyses. We analyzed the differences in epidemiology among distinct groups utilizing the *χ*^2^ test in SPSS Version 23.0 (SPSS Software, Chicago, IL, USA).

## Results

### Literature Retrieval and Study Characteristics

A total of 1788 articles are initially planned to be included in this study, and 1654 were eliminated after screening the titles and abstracts. A total of 108 articles were excluded after more detailed evaluation. As a result, 26 articles (34 studies) [17–42] were brought into this meta-analysis.

The characteristics of the 34 joined studies are listed in Table 1, based on general population samples. The total number of participants in the included studies was 72,319, from 16 provinces of mainland China, and including 18,189 cases with TNs after a diagnosis with ultrasound.

**Table 1:** Characteristics of studies on the morbidity of TNs

First author	Publication year	Location	Rural/urban	Inland/coastal	Study year	UIC (ug/L)	Sample size	Prevalence (%)	case
Yu XH [17]	2008	Panshan, Liaoning	Rural	Inland	1999	103.1	815	12.63	103
Yu XH [17]	2008	Huanghua, Hebei	Rural	Inland	1999	614.6	1056	10.79	114
Yu XH [17]	2008	Zhangwu, Liaoning	Rural	Inland	1999	374.5	1514	10.17	154
Zhu WY [18]	2010	Zhoushan, Zhejiang	Mixed	Coastal	2006	320.7	3284	25.30	831
Lou XM [19]	2011	Xiangshan, etc., Zhejiang	Mixed	Coastal	2009	275.6	280	21.07	59
Lou XM [19]	2011	Haining, etc., Zhejiang	Mixed	Coastal	2009	256.1	321	14.95	48
Lou XM [19]	2011	Daishan, etc., Zhejiang	Mixed	Coastal	2009	149.1	456	14.47	66
Shao HJ [20]	2016	Weihai, Shandong	Rural	Coastal	2009	120.0	835	40.11	335
Liu Y [21]	2012	Chengdu, Sichuan	Urban	Inland	2009	184	1500	17.00	255
Yang YX [22]	2011	Guiyang, Guizhou	Urban	Inland	2009	228.73	1512	10.12	153
Zou SR [23]	2012	Shanghai	Mixed	Coastal	2009	146.7	7369	27.29	2011
Shen Y [24]	2013	Shanghai	Mixed	Coastal	2010	122.8	695	22.88	159
Yang NZ [25]	2012	Taizhou, Zhejiang	Mixed	Inland	2010	178.25	793	22.95	182
Chen ZX [26]	2013	Hangzhou, Zhejiang	Mixed	Inland	2010	172.6	9412	29.98	2822
Zhao XF [27]	2015	Ningbo, Zhejiang	Rural	Coastal	2011	90.4	1177	19.88	234
Du Y [28]	2014	Shuozhou, Shanxi	Mixed	Inland	2012	228.7	531	8.66	46
Du Y [28]	2014	Beihai, Guangxi	Mixed	Inland	2012	62.0	636	22.17	141
Du Y [28]	2014	Taiyuan, Shanxi	Mixed	Inland	2012	750.2	930	15.52	142
Bao CH [29]	2014	Xiangshan, Zhejiang	Mixed	Coastal	2012	140.1	2463	43.80	1079
Meng H [30]	2015	Lishui, Zhejiang	Mixed	Inland	2013	162.7	827	20.31	168
Guo YY [31]	2016	Urumqi, Xinjiang	Urban	Inland	2013	133.4	1835	27.73	509
Gu F[32]	2016	Jiaxing, etc., Zhejiang	Mixed	Inland	2013	180.0	7527	20.59	1550
Gu F [32]	2016	Hangzhou, etc., Zhejiang	Mixed	Coastal	2013	152.0	7568	21.27	1610
Xu FF [33]	2016	Ningbo, Zhejiang	Mixed	Coastal	2014	201.7	913	20.70	189
Wu SB [34]	2018	Huizhou, Guangdong	Mixed	Coastal	2015	149. 25	896	41.96	376
Jing GJ [35]	2020	Longnan, Gansu	Rural	Inland	2015	247.7	1289	16.66	214
Yang WQ [36]	2018	Yinchuan, Ningxia	Urban	Inland	2015	347.6	1292	33.20	429
Lian LX [37]	2018	Harbin, Heilongjiang	Urban	Inland	2015	159.8	2552	48.75	1244
Hu YY [38]	2018	Changde, etc., Hunan	Mixed	Inland	2015	173.9	2650	13.77	365
Song Jun [39]	2016	Shanghai	Mixed	Coastal	2015	132.5	5144	27.76	1428
Cao C [40]	2018	Lanzhou, Gansu	Urban	Inland	2016	205.4	647	21.02	136
Yi JP [41]	2018	Zhoushan, Zhejiang	Mixed	Coastal	2016	126.0	1382	22.72	314
Nima YZ [42]	2018	Lhasa, Tibet	Rural	Inland	2017	140.4	383	38.64	148
Nima YZ [42]	2018	Lhasa, Tibet	Urban	Inland	2017	158.0	1835	31.33	575

### Pooled Morbidity of Thyroid Nodules

**Fig. 2:**
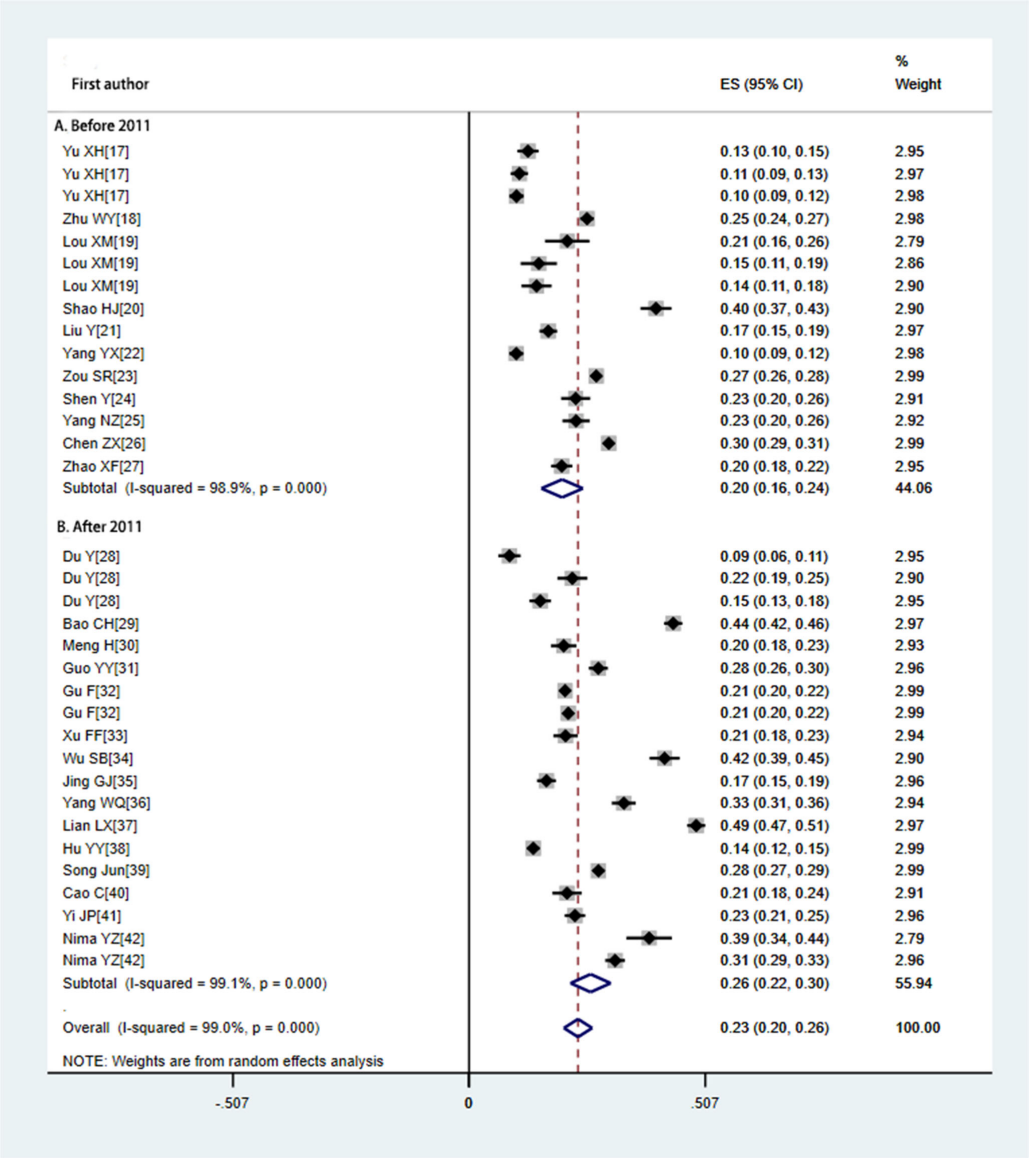
Forest plot of the pooled morbidity of TNs in mainland China

As shown in Fig. 2, averaged over the past two decades, the overall morbidity of TNs in mainland China was 23.4% (95% CI: 20.4–26.4%). The pooled morbidity of TNs before 2011 was 20% (95% CI: 15.5–24.4%); after 2011, the pooled morbidity was 26.1% (95% CI: 21.8–30.4%).

The subgroup morbidity of TNs in mainland China was analyzed as shown in Table 2. The pooled morbidity was higher in urban areas (*χ*^2^ = 351.88, *P* < 0.001) than in rural and mixed areas. Residence in a coastal area (*χ*^2^ = 429.62, *P* < 0.001), female gender (*χ*^2^ = 671.85, *P* < 0.001), residence at high altitude (*χ*^2^ = 56.953, *P* < 0.001), and residence in southern China (*χ*^2^ = 173.86, *P* < 0.001) might indicate higher morbidity of TNs.

**Table 2:** Morbidity of TNs in mainland China by different stratification factors

Subgroups	Prevalence% (95% CI)	Number of studies	Heterogeneity		Case/total
			I^2^%	*P* value	
**Urban/rural**					
Rural	0.211 (0.142–0.279)	7	98.5	< 0.001	1302/7069
Mixed	0.229 (0.198–0.260)	20	98.6	< 0.001	13,586/54077
Urban	0.270 (0.165–0.376)	7	99	< 0.001	2893/8161
**Coastal/inland**					
Coastal	0.261 (0.223–0.298)	14	98.2	< 0.001	4996/18366
Inland	0.215 (0.172–0.259)	20	99.2	< 0.001	9450/22934
**Altitude**					
< 200	0.240 (0.205–0.276)	24	99.1	< 0.001	15,582/60565
200–500	0.17 (0.151–0.189)	1	/	/	255/1500
500–1000	0.215 (0.093–0.337)	2	98.8	< 0.001	651/2765
> 1000	0.227 (0.145–0.309)	7	99	< 0.001	1701/7489
**Iodine status**					
Insufficient	0.207 (0.186–0.229)	2	22.4	0.256	365/1844
Adequate	0.272 (0.235–0.310)	20	99.1	< 0.001	15,299/56937
More than adequate	0.160 (0.120–0.200)	7	94.2	< 0.001	845/5493
Excess	0.189 (0.107–0.272)	5	99	< 0.001	1670/8076
**Region**					
North China	0.177 (0.05–0.304)	6	99.6	< 0.001	1803/7398
South China	0.321 (0.127–0.515)	2	98.6	< 0.001	517/1532
East China	0.246 (0.217–0.274)	17	98.1	< 0.001	13,085/50446
West China	0.243 (0.177–0.310)	8	98.6	< 0.001	2419/10293
Central China	0.138 (0.127–0.515)	1	/	/	365/2650
**Gender**					
Male	0.180 (0.144–0.215)	34	98.5	< 0.001	6129/32502
Female	0.276 (0.237–0.315)	34	98.4	< 0.001	12,060/40867
**Total**	0.234 (0.204–0.264)	34	99	< 0.001	18,189/72319

In the four levels of iodine intake, the lowest level of morbidity of TNs was in the more-than-adequate iodine subgroup, 16% (95% CI: 12–20%). The highest level of morbidity was in the adequate iodine subgroup, 27.2% (95% CI: 23.531%).

**Fig. 3:**
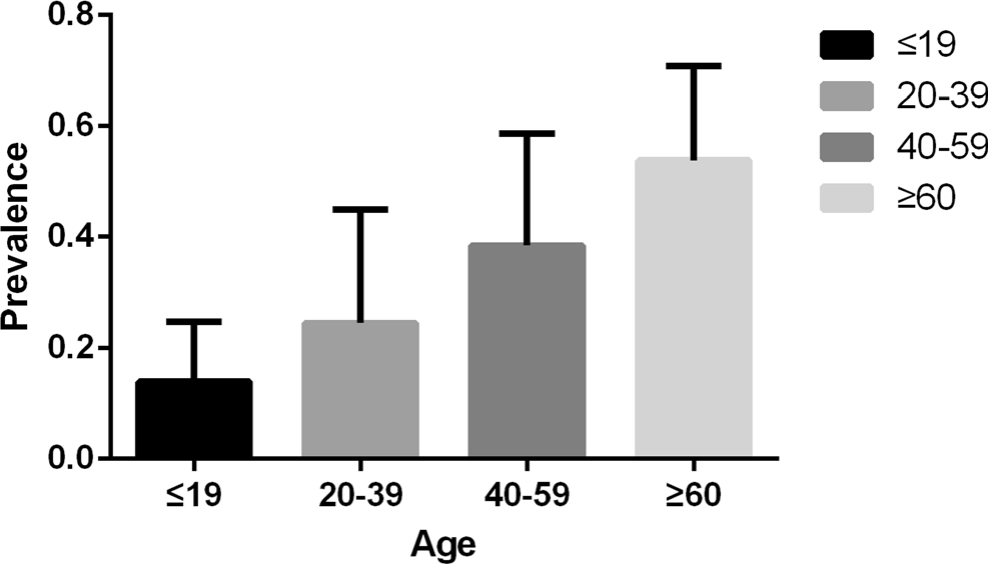
Morbidity of TNs with different ages

**Fig. 4:**
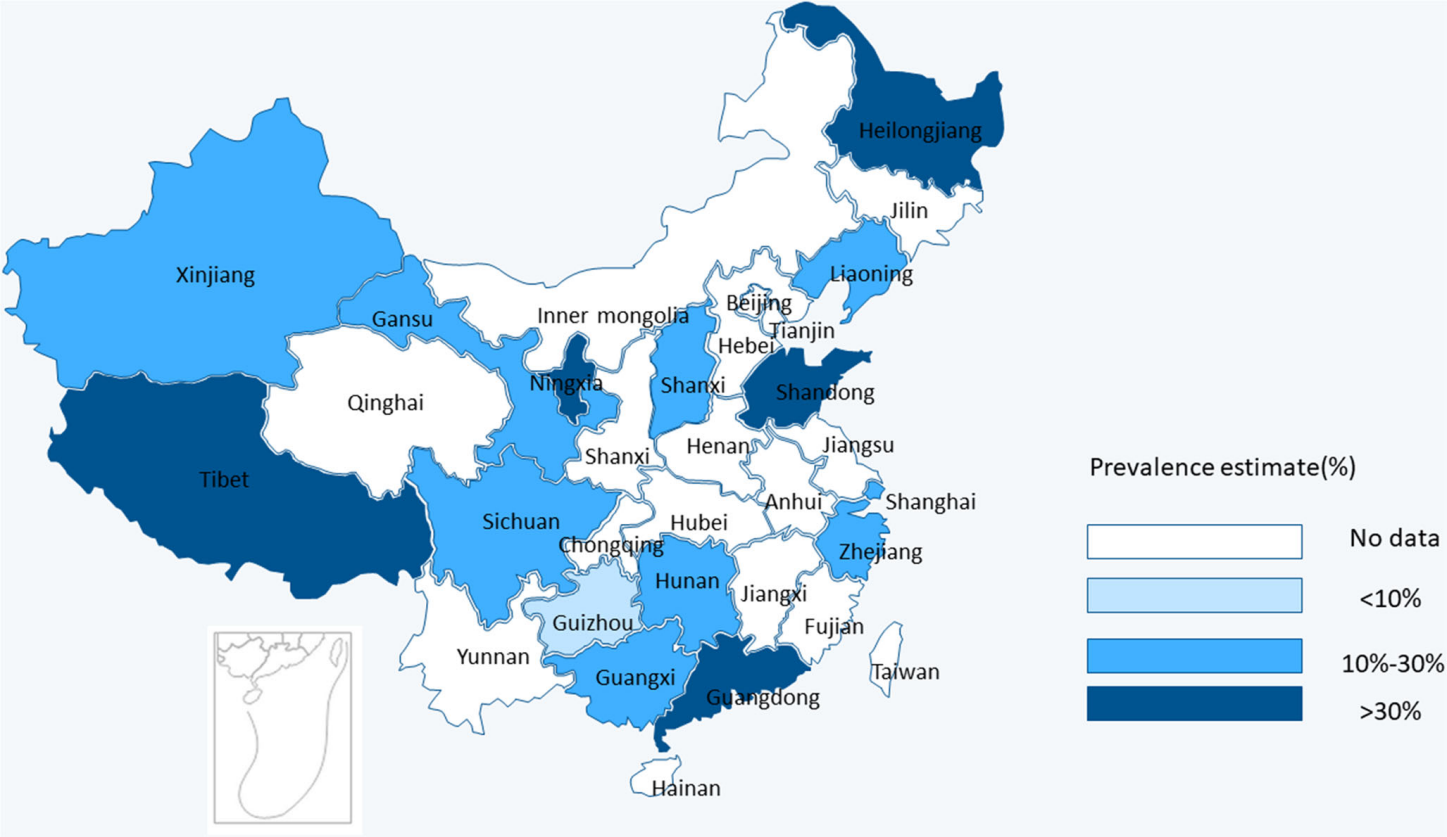
Regional distribution of pooled morbidity of TNs in mainland China

As shown Fig. 3, the morbidity of TNs increased with age, and the highest, 53.3%, was in the group over 60 years old (95% CI: 42.5–64.2%). The lowest morbidity of TNs, 13.8%, was observed in the under-20-year-old group (95% CI: 7.2–20.4%). The morbidity of TNs was 24.2% in the 21–40-year-old group (95% CI: 16.7–31.7%) and 0.38.3% in the 41–61-year-old group (95% CI: 28.7–42.9%). The conditions of the provinces and municipalities are shown in themaps in Fig 4.

### Evaluation of Potential Bias

**Fig. 5:**
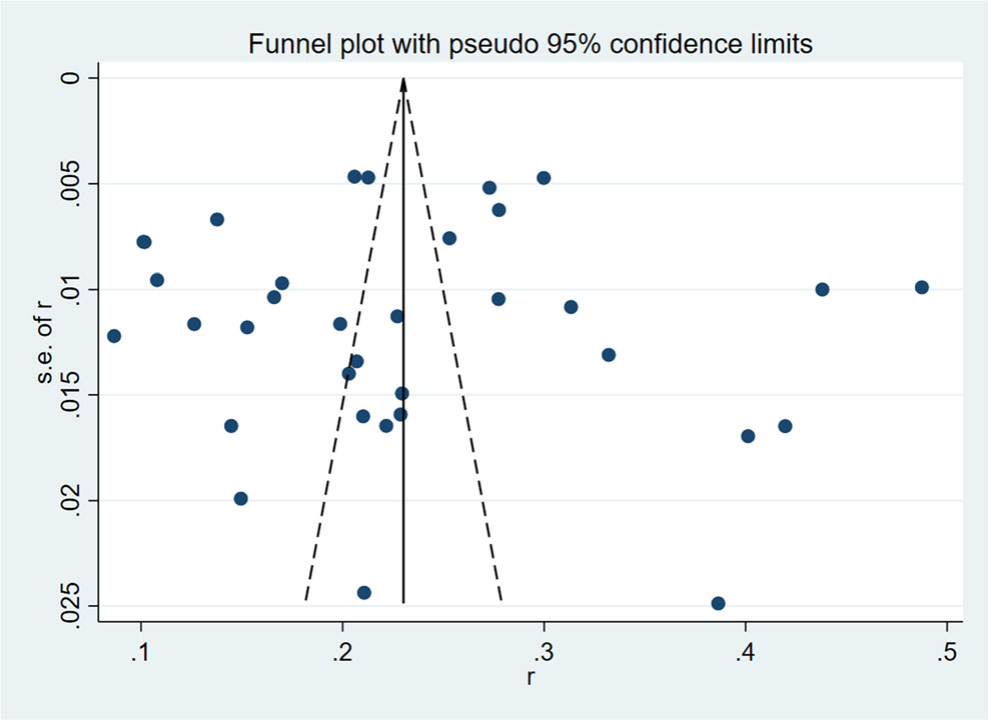
Funnel plot with pseudo 95% confidence limits

For the estimated overall incidence of TNs, we did not detect any significant publication bias using the Egger’s test. The funnel plot was shown in Fig 5.

## Discussion

Thyroid nodules are frequently found in the thyroid gland. Because small nodules can be detected by ultrasound, the morbidity of TNs has been reported as high as 67% in the general population [43]. Because approximately 10–15% of nodules are cancerous, TNs should be observed closely in clinical practice [44, 45].

In our study, on the basis of the inclusion and exclusion criteria, a total of 26 original epidemiological studies were included in this study. After the merger, the sample size reached 18,189 cases and 72,319 patients with TNs, covering 16 provinces. Through a systematic review and meta-analysis of these previous works, an updated estimate of the overall morbidity of TNs was obtained.

The morbidity of TNs in China has been rising over the past 20 years, from 2.73% in 1999 to 17.50% in 2010, and the morbidity rate in 2017 reached 20.43% [46]. The results of our meta-analysis revealed that the morbidity of TNs in mainland China was 23.4%. In the years 2012–2020, the morbidity of TNs was greater than in the years 1999–2011. Teng et al. showed that the morbidity of TNs was 20.43%, which was very high. Furthermore, they also determined that the morbidity of TNs decreased with increases in iodine intake, suggesting that insufficient iodine is a risk factor for TNs and that adequate iodine and more-than-adequate iodine are protective factors for TNs [15]. They supported the conclusion that AI and MAI could be merged as an indicator of an optimal iodine intake for the general population, that is, iodine intake within the range of 100–299 μg/L. But the WHO reported that the optimal urinary iodine concentration was 100–200 μg/L, corresponding approximately to a daily intake of 150–300 μg for adults. We find the optimal median range of urinary iodine recommended by the WHO may need to be broadened. The incidence of TNs and iodine intake formed U-shaped curves, indicating that insufficient and excessive iodine intake both increase the morbidity of TNs. A study from South Korea showed that when the range of UIC was 100–199 μg/L, the morbidity rate of TNs was highest, reaching 19.4%, while the morbidity of TNs decreased with the increase of iodine intake, which further proves that the more-than-adequate iodine is the protective factor of thyroid nodules [47]. In our study, more-than-adequate iodine intake had the lowest morbidity of TNs. Our results are consistent with those of previous studies [15, 39].

Several articles have shown that high-resolution thyroid ultrasound can allow discovery of TNs in 19–68% of stochastically specific individuals, with more women and the elderly suffering from TNs [7, 48]. In our study, a subgroup analysis was carried out by gender, and the morbidity of TNs in males was 18% and 27.6% in females. Women were found to be more likely to experience higher morbidity of TNs than men, which is consistent with the results of earlier studies [49–51]. Possible reasons for this are as follows: (1) thyroid growth factors are susceptible to sex hormones, such as estrogen [52] and 17 beta-estradiol, so women are more likely to be diagnosed with thyroid disease than men [53]. (2) It may also be related to the increased demand for thyroxine during menstruation, pregnancy, and other factors that lead to periodic endocrine changes [54]. According to the subgroup analysis of different ages, as age increases, the morbidity of TNs gradually increases. This may be related to hypothyroidism, dyslipidemia, hormone levels, and other factors. It also indicates that age is one of the factors affecting the occurrence of TNs [55]. We also found high altitude, urban location, and coastal location to be correlated with TNs. Plateau and coastal environments were found to be associated with higher rates of TNs. This finding is similar to that observed for previous studies, and it may be caused by insufficient iodine intake at high altitudes. Excessive iodine in coastal areas necessitates education and economic remedies for the residents who live there [56, 57].

There are two limitations to this research. First, the studies included here covered 16 provinces, mainly in eastern China. Second, we rightly limited the research to mainland China, considering no other countries.

## Conclusion

Our research shows that it is beneficial to formulate iodized salt standards according to local conditions, but there may be some dangerous factors, and these must be considered with care. We need to perform more epidemiological studies, and in the future, we should develop further understanding of the relationship between other thyroid diseases and provide more comprehensive and useful information for other researchers.

## Data Availability

All data used during the study appear in the submitted article and availability.
